# Supplementary material for: A taxonomic review and revisions of Microstomidae (Platyhelminthes: Macrostomorpha)
Source: PLoS One. 2019 Apr 24;14(4):e0212073. doi: 10.1371/journal.pone.0212073 (PMC6481776; doi:10.1371/journal.pone.0212073)
Supplement: S1 Table — Collecting location, specific coordinates and dates are given for each specimen, where available. In addition, the GenBank Accession Numbers for 18S, and CO1 sequences and references are listed. (DOCX) [file pone.0212073.s004.docx]

Table S1. Collecting information for the specimens used in this study. Collecting location, specific coordinates and dates are given for each specimen, where available. In addition, the GenBank Accession Numbers for 18S, and CO1 sequences and references are listed.

Location Specimens Coordinates Date Genbank Accession Numbers Reference

18S COI

*Acanthomacrostomum* sp.

Bocos del Toro, Panama DNA105907 KC869788 Laumer & Giribet 2014

*Austromacrostomum arumoidicornum*

Sant Andrea Bay, Italy MTP LS 638 42°48’31 N 10°08’30 E 29-4-2010 KP730495 KP730571 Janssen et al. 2015

*Bradynectes sterreri*

Sylt, Germany MTP LS 180 55°00’54 N 8°26’16 E 12-03-2007 FJ715298 Scharer et al. 2011

Sylt, Germany MTP LS 162, 5 55°00’54 N 8°26’16 E 10-03-2007 KP730507, 15 KP730567 Janssen et al. 2015

*Cylindromacrostomum* cf. *notandum*

Fetovia Bay, Italy MTP LS 250 42°43’59 N 10°09’14 E 01-05-2007 KP730488 KP730560 Janssen et al. 2015

*Cylindromacrostomum riegeri*

Lignano, Italy MTP LS 546 45°39’18 N 13°06’18 E 22-05-2009 KP730485 KP730583 Janssen et al. 2015

*Dolichomacrostomum uniporum*

Sylt, Germany MTP LS 222 55°02’49 N 8°25’12 E FJ715295 Scharer et al. 2011

*Haplopharynx* cf. *quadristimulus* Sp. C

Bocas del Toro, Panama DNA106018 KC869787 Laumer & Giribet 2014

*Haplopharynx papii*

Sant Andrea Bay, Italy MTP LS 720 42°48’32 N 10°08’31 E 16-06-2015 KP730502 Janssen et al. 2015

*Haplopharynx rostratus*

Malmon, Sweden MTP LS 591 58°20’20 N 11°20’27 E 09-09-2007 KP730509 KP730579 Janssen et al. 2015

AJ012511 Littlewood et al. 1999

*Karlingia lutheri*

Tramanda, Brazil MTP LS 806 29°58’41 S 50°07’12 W 25-06-2010 KP730490 KP730559 Janssen et al. 2015

*Hoploplana californica*

DNA106152 KC869797 Laumer & Giribet 2014

*Macrostomum acus*

China ZDK1-3 KY579351-3 Lin (unpub.)

*Macrostomum balticum*

Sylt, Germany MTP LS 144 54°50’49 N 8°17’53 E 07-03-2007 FJ715310 Scharer et al. 2011

*Macrostomum chongqingensis*

China CQ18S1, 3 KX769148-9 Lin et al. 2017

*Macrostomum clavituba*

Marano, Italy MTP LS 301 45°45’22 N 13°09’54 E 21-07-2007 FJ715304 Scharer et al. 2011

*Macrostomum dongyuanensis*

China DYDK1-2 KY652216-7 Lin (unpub.)

*Macrostomum finlandense*

Schwarzsee, Austria MTP LS 91 47°27’22 N 12°21’58 E 04-07-2006 FJ715302 Scharer et al. 2011

*Macrostomum gieysztori*

Andalucia, Spain MTP LS 264 36°37’37 N 5°10’26 W 03-07-2007 FJ715301 Scharer et al. 2011

*Macrostomum heyuanensis*

China KX755246 Sun et al. 2015

*Macrostomum hystricinum*

AF051329 Ruiz-Trillo et al. (unpub.)

*Macrostomum hystricinum marinum*

Grado, Italy MTP LS 278 45°42’50 N 13°23’06 E 17-07-2007 FJ715311 Scharer et al. 2011

*Macrostomum hystrix*

Bibione, Italy MTP LS 68 45°38’49 N 13°01’12 E 11-04-2006 FJ715303 KP730561 Scharer et al. 2011

*Macrostomum kepneri*

Bibione, Italy MTP LS 285 45°38’49 N 13°01’12 E 18-07-2007 FJ715307 Scharer et al. 2011

*Macrostomum lignano*

Cultured MTP LS 244 45°42’14 N 13°09’29 E FJ715306 KP730568 Scharer et al. 2011

*Macrostomum longituba*

Bibione, Italy MTP LS 274 45°38’49 N 13°01’12 E 17-07-2007 FJ715309 Scharer et al. 2011

*Macrostomum mystrophorum*

Bibione, Italy MTP LS 64 45°39’18 N 13°04’12 E 10-04-2006 FJ715305 Scharer et al. 2011

*Macrostomum obtusa*

China DDK1-3 KY579348-50 Lin (unpub.)

*Macrostomum pusillum*

Lignano, Italy MTP LS 112 45°41’31 N 13°07’51 E FJ715313 Scharer et al. 2011

U. Basel, Switzerland MTP LS 132 45°50’53 N 8°17’53 E 07-03-2007 FJ715314 KP730558 Scharer et al. 2011

*Macrostomum quiritum*

U. Basel, Switzerland MTP LS 102 47°33’32 N 7°34’55 E 09-11-2006 FJ715299 Scharer et al. 2011

*Macrostomum rubrocinctum*

Massachusetts, USA DNA105928 41°44’01 N 70°22’49 W KC869789 Laumer & Giribet 2014

*Macrostomum sinensis*

China ZGDK1-3 KY579354-6 Lin (unpub.)

*Macrostomum spirale*

Sylt, Germany MTP LS 227 54°50’49 N 8°17’53 E FJ715308 KP730565 Scharer et al. 2011

*Macrostomum tuba*

Ushimado, Okayama, Japan D85091 Katayama et al. 1996

U. Basel, Switzerland MTP LS 261 47°33’32 N 7°34’55 E 26-06-2007 FJ715300 Scharer et al. 2011

Mississippi, USA MTP LS 586 34°03’54 N 88°25’01 W 07-10-2009 KP730496 KP730586 Janssen et al. 2015

U70080 Carranza et al. 1997

*Macrostomum zhaoqingensis*

China ZQ18s1-2 KX769146-7 Lin et al. 2017

*Macrostomum zhujiangensis*

China Isolate 1-3 KX771196-8 Fang et al. 2016

*Microstomum afzelii* sp. nov.

Saltö, Sweden J09 58°52’29 N 11°08’41 E 18-06-2016 MK504480 MK504554

*Microstomum artoisi*

Hoge Kempen Park, Belgium B01 51°00’03 N 05°40’44 E 20-06-2017 MH221275 MH221383 Atherton & Jondelius 2018b

Campus Pond, Belgium B02 50°57’45 N 05°23’23 E 20-06-2017 MH221276 MH221384 Atherton & Jondelius 2018b

*Microstomum bispiralis*

Sundsvallen, Sweden A06 63°24’01 N 12°49’51 E 03-07-2016 MK504481

Tångböle, Sweden A09 63°21’42 N 12°40’40 E 04-07-2016 MK504482 MK504555

Dorotea, Sweden A12-16,18 64°15’42 N 16°14’26 E 07-07-2016 MK504483-6 MK504556-61

Sorsele, Sweden A21 65°29’07 N 17°33’37 E 08-07-2016 MK504562

Aha, Sweden A22-24,26 65°27’33 N 17°46’19 E 09-07-2016 MK504563-6

Bure, Sweden A33 65°33’24 N 17°51’44 E 12-07-2016 MK504567

Sorsele, Sweden A40 65°32’21 N 17°38’26 E 14-07-2016 MK504568

Slagnäs, Sweden A43 65°35’21 N 18°09’47 E 14-07-2016

Pirttivuopio, Sweden A45-50,52-54 67°52’11 N 19°13’07 E 14-07-2016 MK504487-91 MK504569-73

Järnäshamn, Sweden A121 63°26’26 N 19°39’13 E 29-07-2016 MK504492

Malmö, Sweden C03-04 55°36’14 N 12°58’05 E 03-09-2015 MK504493 MK504574

Malmö, Sweden C16-18 55°40’16 N 13°03’42 E 04-09-2015 MK504494 MK504575-6

Ivösjön, Sweden C75 56°04’04 N 14°27’13 E 06-09-2015 MK504495 MK504577

Ekerö, Sweden E39-40,44-49 59°16’21 N 17°44’18 E 10-05-2015 MK504578-85

Ekerö, Sweden E52-57,59-61 59°16’21 N 17°44’18 E 10-05-2015 MK504496-9 MK504586-93

Blackberg, Sweden E64, E78-9 59°20’12 N 17°53’54 E 10-06-2015 MK504500

Fjäturen, Finland E120-1, 59°27’33 N 18°00’06 E 26-07-2017

Ymsen, Finland E133,135,137-9 58°42’22 N 14°00’25 E 29-07-2017

Mustalahti, Finland F03-6 62°12’47 N 24°45’39 E 07-06-2017 MK504501-4

Ratalahti, Finland F12-14,16-21 61°02’40 N 23°26’52 E 07-06-2017 MK504505-13

Paattistenjoki, Finland F22-24 60°31’57 N 22°21’12 E 08-06-2017 MK504514-6

Sahinlampi, Finland F25 62°05’39 N 24°43’40 E 09-06-2017 MK504517

Haunisten Allas, Finland F29 60°29’58 N 22°12’17 E 12-06-2017 MK504518

Gotland, Sweden G17 57°54’05 N 18°50’14 E 14-08-2016 MK504519 MK504595

Gotland, Sweden G22,26 57°51’03 N 18°48’52 E 16-08-2016 MK504520-1 MK504596

Saltö, Sweden J02-4 58°52’29 N 11°08’41 E 16-06-2016 MK504522-4

Kurland, Sweden K25 55°22’28 N 13°03’51 E 23-05-2016 MK504525 MK504598

Ystad, Sweden K44-46 55°25’43 N 13°51’32 E 25-05-2016 MK504599-601

*Microstomum compositum*

Fiskebäckskil, Sweden S44,78-82 58°15’00 N 11°26’45 E 23-08-2015 MK504476-9 MK504602-7

*Microstomum crildensis*

Tjärnö, Sweden J12-13 58°53’02 N 11°08’28 E 19-06-2016 MK504526-7 MK504608-9

Malmön, Sweden S03 58°20’40 N 11°20’56 E 14-08-2015 MK504528 MK504610

Fiskebäckskil, Sweden S53 58°15’00 N 11°26’45 E 20-08-2015 MK504529

*Microstomum curinigalletti* sp. nov.

Fiskebäckskil, Sweden S25,27-8,56,58 58°14’49 N 11°26’45 E 21-08-2015 MK504530-4

*Microstomum edmondi*

Munkedal, Sweden S17, S30 58°27’31 N 11°41’10 E 15-08-2015 MH221282-3 MF185701-02 Atherton & Jondelius 2018a

Munkedal, Sweden S5,11-20,31-2 58°27’31 N 11°41’10 E 15-08-2015 MK504535-9 MK504611-21

*Microstomum inexcultus* sp. nov.

Saltö, Sweden J5-6, 8, 11 58°52’29 N 11°08’41 E 18-06-2016 MK504540 MK504622-5

Tjärnö, Sweden J15-16 58°53’02 N 11°08’28 E 19-06-2016 MK504541 MK504626-7

Malmön, Sweden S04 58°20’41 N 11°20’56 E 14-08-2015 MK504542 MK504628

Fiskebäckskil, Sweden S66 58°15’05 N 11°27’52 E 22-08-2015 MK504543 MK504629

*Microstomum laurae*

Saltö, Sweden J10 58°52’41 N 11°06’56 E 19-06-2016 MH221284 MF185712 Atherton & Jondelius 2018a

Saltö, Sweden J18-2 58°52’29 N 11°08’41 E 17-06-2016 MH221285 MF185713 Atherton & Jondelius 2018a

*Microstomum lineare*

Tensjön, Sweden A02, A03 61°39’36 N 15°14’52 E 01-07-2016 MH221198-9 MF185698-9 Atherton & Jondelius 2018a

Gevsjön, Sweden A04, A05 63°21’42 N 12°40’40 E 02-07-2016 MH221200-1 MH221288-9 Atherton & Jondelius 2018b

Ängermanälven, Sweden A19 64°26’03 N 16°47’16 E 06-07-2016 MH221202 MH221290 Atherton & Jondelius 2018b

Grundträsket, Sweden A27 65°28’29 N 17°37’45 E 08-07-2017 MH221203 MH221291 Atherton & Jondelius 2018b

Ahasjön, Sweden A30 65°27’33 N 17°46’19 E 08-07-2017 MH221204 MH221292 Atherton & Jondelius 2018b

Norr-svergoträsket, Sweden A31-4,36-8,41-2 65°32’21 N 17°38’26 E 10-07-2016 MH221205-12 MH221293-300 Atherton & Jondelius 2018b

Buresjön, Sweden A39 65°33’24 N 17°51’44 E 10-07-2016 MH221213 MH221301 Atherton & Jondelius 2018b

Pirttivuopio, Sweden A51,56-7 67°52’11 N 19°13’07 E 13-07-2016 MH221214-6 MH221302-3 Atherton & Jondelius 2018b

Alavuopio, Sweden A58 67°52’06 N 19°16’52 E 13-07-2016 MH221217 MH221304 Atherton & Jondelius 2018b

Laukkujärvi, Sweden A59 67°49’49 N 19°37’22 E 13-07-2016 MH221218 MH221305 Atherton & Jondelius 2018b

Pond by E10, Sweden A60 68°20’53 N 18°58’12 E 15-07-2016 MH221219 MH221306 Atherton & Jondelius 2018b

Creek by E10, Sweden A61-4 68°25’41 N 18°26’44 E 17-07-2016 MH221220-3 MH221307-10 Atherton & Jondelius 2018b

Báktájävri, Sweden A69-70,73-4 68°25’51 N 18°33’16 E 17-07-2016 MH221224-6 MH221311-4 Atherton & Jondelius 2018b

Stor-Tannörsavan, Sweden A107-8,120 63°26’26 N 19°39’13 E 28-07-2016 MH221227-9 MH221315-7 Atherton & Jondelius 2018b

Lilla-Tannörsavan, Sweden A116,122,135 63°26’27 N 19°38’42 E 28-07-2016 MH221230-1 MH221318-20 Atherton & Jondelius 2018b

Bergsjön, Sweden A129-31, 33-4 63°38’27 N 19°10’37 E 31-07-2016 MH221232-3 MH221321-5 Atherton & Jondelius 2018b

Yttre Lemesjön, Sweden A136-7 63°37’53 N 19°03’37 E 31-07-2016 MH221234 MH221326-7 Atherton & Jondelius 2018b

Storsjön, Sweden A138 63°37’44 N 18°41’15 E 31-07-2016 MH221328 Atherton & Jondelius 2018b

Campus Pond, Belgium B03-4 50°57’45 N 05°23’23 E 20-06-2017 MH221235-6 MH221329-30 Atherton & Jondelius 2018b

Hoge Kempen Park, Belgium B21 51°00’03 N 05°40’44 E 20-06-2017 MH221237 MH221331 Atherton & Jondelius 2018b

Valjeviken, Sweden E56,62 56°03’44 N 14°32’25 E 05-09-2015 MH221238 MH221332-3 Atherton & Jondelius 2018b

Mälaren, Sweden E63-8 59°20’12 N 17°53’54 E 06-10-2015 MH221239-42 MH221334-9 Atherton & Jondelius 2018b

Rosjön, Sweden E90-1,95,104 59°26’26 N 18°00’03 E 27-07-2017 MH221243-6 MH221340-3 Atherton & Jondelius 2018b

Edsviken, Sweden E102-3 59°25’47 N 17°57’42 E 30-07-2017 MH221247-8 MH221344-5 Atherton & Jondelius 2018b

Mariestadssjön, Sweden E105-19,22,6-30 58°42’59 N 13°50’04 E 30-07-2017 MH221249-57 MH221346-67 Atherton & Jondelius 2018b

Riitalahti, Finland F02,10 62°12’47 N 24°45’39 E 05-06-2017 MH221258-60 MH221368-70 Atherton & Jondelius 2018b

Mustalahti, Finland F08 62°01’31 N 24°41’03 E 05-06-2017 MH221261 MH221371 Atherton & Jondelius 2018b

River in Loimaa, Finland F09 60°54’58 N 23°12’13 E 05-06-2017 MH221262 MH221372 Atherton & Jondelius 2018b

Haunisten allas, Finland F26-35 60°29’58 N 22°12’17 E 10-06-2017 MH221263-70 MH221373-80 Atherton & Jondelius 2018b

Tingstädeträsk, Sweden G16,19 57°44’18 N 18°37’32 E 09-08-2016 MH221671-2 Atherton & Jondelius 2018b

Pond in Lärbro, Sweden G23-4 57°51’03 N 18°48’52 E 15-08-2016 MH221273-4 MH221381-2 Atherton & Jondelius 2018b

Läppträsket, Finnland MTP LS 394 60°03’07 N 23°40’19 E 06-08-2008 KP730484 KP730567 Jannsen et al. 2015

Connecticut, USA DNA105906 01-09-2009 KC869791 Laumer & Giribet 2014

Ushimado, Okayama, Japan D85092 Katayama et al. 1996

United Kingdom AJ405979 Telford et al. 2000

*Microstomum lotti* sp. nov.

Sant Andrea Bay, Italy MTP LS 660,635 42°48’31 N 10°08’30 E 26-04-2010 KP730483, 93 Jannsen et al. 2015

*Microstomum* *marisrubri* sp. nov.

Mangrove Bay, Egypt MTP LS 524 25°52’15 N 34°25’04 E 11-01-2009 KP730505 KP730580 Jannsen et al. 2015

*Microstomum papillosum*

Koenighafen, Germany MTP LS 146 55°02’24 N 08°23’52 E 08-03-2007 FJ715296 KP730570 Jannsen et al. 2015

*Microstomum rubromaculatum*

Fiskebäckskil, Sweden S35-37 58°14’59 N 11°26’45 E 20-08-2015 MH221286-7 MF185693-5 Atherton & Jondelius 2018a,b

Fiskebäckskil, Sweden S38-42,46,50-1 58°14’59 N 11°26’45 E 20-08-2015 MK504546-9 MF185686-92 Atherton & Jondelius 2018a

Fiskebäckskil, Sweden S34,58,65,83 58°14’59 N 11°26’45 E 20-08-2015 MK504550-1 MF185684-5,96 Atherton & Jondelius 2018a

*Microstomum schultei* sp. nov.

Fetovaia Bay, Italy MTP LS 700 42°43’36 N 10°09’33 E 26-04-2010 KP730494 Jannsen et al. 2015

*Microstomum septentrionale*

Fiskebäckskil, Sweden S111-113 58°15’05 N 11°27’52 E 25-08-2015 MK504552 MK504630-2

*Microstomum tchaikovskyi*

Rosjön, Sweden E83 59°26’26 N 18°00’03 E 15-09-2016 MH221277 MH221385 Atherton & Jondelius 2018b

Mustalahti, Finland F07 62°01’31 N 24°41’03 E 05-06-2017 MH221278 MH221386 Atherton & Jondelius 2018b

*Microstomum weberi* sp. nov.

Pianosa, Italy MTP LS 666 42°34’29 N 10°03’59 E 30-04-2010 KP730487 KP730576 Jannsen et al. 2015

*Microstomum westbladi* nom. nov.

Fiskebäckskil, Sweden S114 58°15’05 N 11°27’52 E 25-08-2015 MK504553 MK504633

*Microstomum zicklerorum*

Massachusetts, USA L01-3 42°38’13 E 71°23’31 W 11-04-2017 MH221279-81 MH221387-9 Atherton & Jondelius 2018b

*Myomacrostomum rubrioculum*

Pianosa, Italy MTP LS 670 42°34’30 N 10°03’58 E 02-05-2010 KP730499 Jannsen et al. 2015

*Myozonaria bistylifera*

Fetovaia Bay, Italy MTP LS 632 42°43’36 N 10°09’33 E 26-04-2010 KP730486 KP730573 Jannsen et al. 2015

Sant Andrea Bay, Italy MTP LS 671 42°48’31 N 10°08’33 E 26-04-2010 KP730510 KP730584 Jannsen et al. 2015

*Myozonaria fissipara*

Sant Andrea Bay, Italy MTP LS 623,678 42°48’31 N 10°08’30 E 30-04-2010 KP730497-8 KP730574-5 Jannsen et al. 2015

*Myozona lutheri*

Sant Andrea Bay, Italy MTP LS 692 42°48’32 N 10°08’31 E 04-05-2010 KP730491 KP730582 Jannsen et al. 2015

*Myozona* sp.

Sant Andrea Bay, Italy MTP LS 731 42°48’32 N 10°08’31 E 13-07-2010 KP730501 Jannsen et al. 2015

Myozonariinae

Pianosa, Italy MTP LS 669,673 42°34’29 N 10°03’59 E 30-04-2010 KP730503 KP730572, 69 Jannsen et al. 2015

*Paracatenula* sp.

IZ29193 KC869782 Laumer & Giribet 2014

*Paromalostomum dubium*

Sylt, Germany MTP LS 124 55°00’54 N 8°26’17 E 06-03-2007 KP730489 KP730563 Jannsen et al. 2015

*Paromalostomum fusculum*

Sylt, Germany AJ012531 Littlewood et al. 1999

Sylt, Germany MTS LS 119 55°00’58 N 8°26’17 E 05-03-2007 KP730504 Janssen et al. 2015

*Paromalostomum massiliensis*

Sant Andrea Bay, Italy MTP LS 708 42°48’32 N 10°08’31 E 05-05-2010 KP730506 KP730578 Jannsen et al. 2015

*Paromalostomum minutum*

Lignano, Italy MTP LS 555 45°39’18 N 13°06’22 E 26-05-2009 KP730511 Jannsen et al. 2015

Fetovaia Bay, Italy MTP LS 696 42°43’55 N 10°09’25 E 05-05-2010 KP730512 KP730581 Jannsen et al. 2015

*Psammomacrostomum* sp. 1

Hango, Finland MTP LS 380 59°49’19 N 22°58’19 E 05-08-2008 KP730508 Jannsen et al. 2015

*Psammomacrostomum* sp. 2

Lignano, Italy MTP LS 309 45°41’28 N 13°07’55 E 22-07-2007 KP730481 Jannsen et al. 2015

*Psammomacrostomum* sp. 3

Sant Andrea Bay, Italy MTP LS 624 42°48’32 N 10°08’31 E 27-04-2010 KP730500 Jannsen et al. 2015

*Psammomacrostomum* sp. 4

Pianosa, Italy MTP LS 633 42°35’24 N 10°05’46 E 28-04-2010 KP730514 Jannsen et al. 2015

*Psammomacrostomum* sp. 5

Sant Andrea Bay, Italy MTP LS 719 42°48’32 N 10°08’31 E 06-05-2010 KP730513 KP730585 Jannsen et al. 2015

*Stenostomum* sp.

Öland, Sweden K04_81 09-04-2009 FJ384811 KP730282 Larsson et al. 2008
