# Supplementary material for: A taxonomic review and revisions of Microstomidae (Platyhelminthes: Macrostomorpha)
Source: PLoS One. 2019 Apr 24;14(4):e0212073. doi: 10.1371/journal.pone.0212073 (PMC6481776; doi:10.1371/journal.pone.0212073)
Supplement: S2 Table — (DOCX) [file pone.0212073.s005.docx]

Table S2: Primer sequences, references and protocols for amplification of 18S and CO1 sequences.

Gene

Primer Direction Reference Sequence

Protocol

18S

WormA Forward Littlewood & Olsen, 2001 GCGAATGGCTCATTAAATCAG

WormB Reverse Littlewood & Olsen, 2001 CTTGTTACGACTTTTACTTCC

1270F Internal Littlewood et al. 2000 ACTTAAAGGAATTGACGG

1270R Internal Littlewood et al. 2000 CCGTCAATTCCTTTAAGT

5 min at 94°C; 40x (30s at 94°C, 30s at 54°C, 2 min at 72°C); 10 min at 72°C

CO1

Mac_COIF Forward Janssen et al. 2015 GTTCTACAAATCATAAGGATATTGG

Mac_COIR Reverse Janssen et al. 2015 TAAACYTCWGGGTGACCAAAAAACCA

Mic_37F Forward this paper GTTTGAGGAGGTTTGATAGGGC

Mic_638R Reverse this paper ATTACATCACCCCCTCCCGT

5 min at 94°C; 5x (30s at 94°C, 90s at 45°C, 60s at 72°C); 35x (30s at 94°C, 90s at 51°C, 60s at 72°C); 10 min at 72°C
